# Awareness and interventions to reduce dehydration in pregnant, postpartum women, and newborns in rural Kenya

**DOI:** 10.4102/phcfm.v15i1.3991

**Published:** 2023-05-15

**Authors:** Adelaide Lusambili, Britt Nakstad

**Affiliations:** 1 Institute for Human Development, Faculty of Public Health, The Aga Khan University, Nairobi, Kenya; 2 Environmental Center, Leadership and Governance HUB, School of Business, African International University, Nairobi, Kenya; 3 Department of Pediatric and Adolescent Health, Faculty of Medicine University of Botswana, Gaborone, Botswana; 4 Division of Pediatric and Adolescent Medicine, Faculty of Medicine, Institute of Clinical Medicine, University of Oslo, Oslo, Norway

**Keywords:** dehydration, newborns, pregnant and postpartum women, climate change, heat, drought

## Abstract

Extreme heat exposure is associated with adverse outcomes in pregnancy and has the potential to impact maternal, neonatal and child health for a lifetime. In an extremely hot climate, pregnant women face an increased risk of premature birth, stillbirth, low birth weight, congenital anomalies and pre-eclampsia. In low- and middle-income countries (LMICs), socio-demographic and behavioural practices may negatively affect body hydration during high temperatures. The possible causes and consequences of dehydration in the heat are poorly understood and have been little discussed in the literature.

Living in a hot climate poses various challenges, including dehydration, where biological mechanisms and insufficient access to water can lead to dehydration in women and children, with consequences for the health of both mothers and children, particularly in relation to breastfeeding habits. During pregnancy, increased metabolic and cardiovascular demands interact with heat exhaustion and reduced availability of fresh water, which can affect the child’s growth and development. In this opinion piece, we emphasise the possible causes and impacts of dehydration in extreme heat on the health and well-being of mothers and children. We encourage more research, focused on biology and epidemiology, related to raising awareness and implementing adaptations to reduce the risk of dehydration in pregnant, postpartum women and newborns in the context of climate change-related heat exposure.

## Causes and effects of dehydration

Dehydration has various causes and detrimental effects. The harmful impacts of climate change, including hotter, longer dry spells, affect everyone. However, in rural Kenya, pregnant and postpartum women, as well as their newborns, bear the brunt of heat-related exposure, such as dehydration. High temperature, humidity and environmental drought all pose challenges to accessing enough clean water, which is vital to maintaining an optimal hydration status for good health and physical activity.

During pregnancy, water is needed for building and maintaining foetal growth, development and metabolic activity.^1^ In order for a foetus to develop and grow adequately, the mother must normally drink more water than she did prior to becoming pregnant.^2^ However, research on fluid intake among pregnant women is scarce, with little published scientific literature available on recommended intake for pregnant and lactating women. A study from India, where a cohort of 38 women were followed, found that only about one-third of the women had adequate water intake, with possible consequences on birth weight. Researchers found that only about one-third of the women had adequate water intake.^1^

Bodily water participates in a variety of physiological functions, including those related to pregnancy. Water is needed during pregnancy to support foetal growth, development and metabolic activity.^1^ Total and extracellular water significantly increases as pregnancy progresses,^3^ and this increase is a part of the body’s adaptation to pregnancy.^4^ During pregnancy, there are also significant increases in blood volume and red blood cells, and cardiac output increases. However, some pregnant mothers may experience increased shortness of breath and fatigue.^5^ A water imbalance is even a strong predictor of gestational hypertension,^4^ and preeclampsia,^6^ low birth weight and poor pregnancy outcomes.^3,7^ During pregnancy, water loss through sweating increases because of hyperactive adrenal and thyroid functions, an accelerated metabolism and increased cutaneous circulation. These factors add to the challenges posed by environmental factors such as heat and drought in Kenya. In addition to increasing water intake, nutrients and energy requirements also increase during pregnancy. It is recommended that women increase their fluid intake by at least 300 mL and their caloric consumption by about 300 calories, beginning in the second trimester.^8^

Published literature on neonatal and infant dehydration as a consequence of heat exposure and environmental challenges is scarce.^9^ Dehydration in this vulnerable population is mostly associated with gastroenteritis, pyelonephritis or bronchiolitis.^10^ Insufficient hydration in breastfed newborns is common, and the very hot climate increases the risk of small-sized babies losing too much water and not being able to drink enough milk to replace the loss.^11^ If the mother is dehydrated, her breast milk composition can change and the quantity of milk produced may not be enough for the baby. As a result the baby’s health may be dangerously threatened. Consequences of extreme heat associated with dehydration, starvation and deprivation contribute to fully preventable serious health problems in the mother, overlapping with the riskiest period for sickness and death in her baby.^12,13^

## Water scarcity

Water scarcity is a significant challenge in many parts Kenya especially in arid and semi-arid regions, such as the Coastal, Eastern and North Eastern parts of Kenya. Pregnant and postpartum women, as well as their unborn and newborn children, are particularly affected by this problem.^14^ Women in these areas are often the primary water collectors and have to spend long hours searching for water, sometimes more than 2 hours, to bring back to their family and crops.^14^ Pregnant women in these settings may not even drink the water because of contamination fears, leaving them dehydrated and fatigued. This dehydration can lead to headaches and physical weakness, making it difficult for women to perform their daily tasks, including giving birth. Prolonged labour is common in dehydrated women, often requiring emergency C-sections to save lives. Newborns of dehydrated mothers are nearly certain to arrive sickly, dehydrated and underweight or some dying in the womb or shortly after birth.

## Traditional beliefs

As if the sweltering heat, drought, restrictive dress codes and poor living conditions were not enough, traditional beliefs held by many in rural villages are adding to the plight of mothers and babies. In some cultures, particularly along the coastal and North Eastern regions of Kenya, there are beliefs that drinking water during pregnancy suppresses babies’ activity in the womb, leading to stillbirths. Even nursing mothers are discouraged from drinking water in some communities, as local lore suggests that it freezes breast milk. Even as ambient temperatures reach higher extremes, a lack of water supplies, rampant misinformation and local mores continue to deprive mothers and children of the water they need to survive.

## Indoor environment

In many of the rural areas, houses are small, often built with corrugated iron sheets and no windows.^15^ When women are not out tilling the fields or looking for water, many stay in-doors, preparing and cooking food over open fires. Modesty requirements sometimes necessitate covering their bodies entirely while babies are swathed in many layers of clothes to avoid the harmful effects of the ‘evil eye’. The indoor environment, coupled with the lack of available cleaning water and heavy layering of clothes, may amplify dehydration in pregnant and postpartum women and their newborns. Still, in most of rural Kenya, women strap their babies on their backs and walk with them in the heat while performing outdoor chores, which can lead to the increased loss of fluids in the body.

## Interventions to promote adequate hydration during pregnancy, postpartum and for newborns

Many rural women in Kenya lack knowledge about the importance of hydration. The Ministry of Health needs to make information available at different levels. Healthcare providers who work with women during antenatal and postnatal care can provide information on the importance of hydration, including recommended daily fluid intake. This information can be communicated to mothers in their local languages. Community health volunteers can embed hydration messages in their daily work and impress upon women, during visits, to boil and drink more water. Just as urgently, local leaders, chieftains, mothers-in-laws, grandmothers and traditional birth attendants (TBAs) need to be educated to sensitise their communities about the importance of drinking water for maternal and newborn health.

There is need to raise awareness to counter harmful traditional beliefs that may make women more vulnerable to the heat and worsen their dehydration. Such awareness can stress the benefits of reducing the workload of pregnant and nursing mothers, including reducing the time spent searching for water in the heat. Community members, especially pregnant and postpartum women support networks, could be educated to detect the early signs of dehydration in newborns, pregnant and nursing mothers. Church leaders and local leaders should be educated to include hydration messages in their daily work, such as less common symptoms like headache, dizziness, a lack of focus, light headedness and weakness.

Early warning systems and cost-effective interventions can be developed to reduce the risk of dehydration in the heat and consequently poor health outcomes in these vulnerable groups.

## Improving water sources

In Kenya’s Coastal and ASAL regions, water availability is unpredictable, which exacerbates water scarcity and limits access to clean water. Communities must begin to co-develop solutions and adapt to the effects of extreme weather to protect the health of women and newborns. Governments and relevant ministries must work together to invest in transitioning to solar-powered water systems.

## Future research

As long spells of drought and high-temperature continue to increase, more vulnerable populations such as pregnant women and neonates will suffer from dehydration. Research on the importance of water and hydration in pregnancy and postpartum is scarce and neglected. There is an urgent need for research to examine the fluid intake and hydration status of pregnant women and their newborns and to investigate the associations of complications and maternal–child outcomes. To inform interventions that can improve hydration for diverse women, research should focus on examining the water intake needs of pregnant women on a monthly basis while considering their height and weight. Lastly, more understanding is still needed in the African context to shed light on the effect of hydration on babies’ weight, length and cognitive functioning.
